# Unavoidable Human Errors of Tumor Size Measurement during Specimen Attachment after Endoscopic Resection: A Clinical Prospective Study

**DOI:** 10.1371/journal.pone.0121798

**Published:** 2015-04-09

**Authors:** Hirohito Mori, Hideki Kobara, Takaaki Tsushimi, Noriko Nishiyama, Shintaro Fujihara, Tsutomu Masaki

**Affiliations:** 1 Department of Gastroenterology and Neurology, Kagawa University, Kagawa, Japan; 2 Department of Gastroenterological Surgery, Ehime Rosai Hospital, Niihama, Japan; University Hospital Llandough, UNITED KINGDOM

## Abstract

**Objective:**

Objective evaluation of resected specimen and tumor size is critical because the tumor diameter after endoscopic submucosal dissection affects therapeutic strategies. In this study, we investigated whether the true tumor diameter of gastrointestinal cancer specimens measured by flexible endoscopy is subjective by testing whether the specimen is correctly attached to the specimen board after endoscopic submucosal dissection resection and whether the size differs depending on the endoscopist who attached the specimen.

**Methods:**

Seventy-two patients diagnosed with early gastric cancer who satisfied the endoscopic submucosal dissection expanded-indication guideline were enrolled. Three endoscopists were randomly selected before every endoscopic submucosal dissection. Each endoscopist separately attached the same resected specimen, measured the maximum resection diameter and tumor size, and removed the lesion from the attachment board.

**Results:**

The resected specimen diameters of the 3 endoscopists were 44.5±13.9 mm (95% Confidence Interval (CI): 23–67), 37.4±12.0 mm (95% CI: 18–60), and 41.1±13.3 mm (95% CI: 20–63) mm. Comparison among 3 groups (Kruskal Wallis *H*- test), there were significant differences (*H* = 6.397, *P* = 0.040), and recorded tumor sizes were 38.3±13.1 mm (95% CI: 16–67), 31.1±11.2 mm (95% CI: 12.5–53.3), and 34.8±12.8 (95% CI: 11.5–62.3) mm. Comparison among 3 groups, there were significant differences (*H* = 6.917, *P* = 0.031).

**Conclusions:**

Human errors regarding the size of attached resected specimens are unavoidable, but it cannot be ignored because it affects the patient’s additional treatment and/or surgical intervention. We must develop a more precise methodology to obtain accurate tumor size.

**Trial Registration:**

University hospital Medical Information Network UMIN No. 000012915

## Introduction

Endoscopic submucosal dissection (ESD) for early gastric cancer, which has been covered by insurance since its introduction, has become a standard therapy for early gastric cancer in Japan, and various endoscopic management techniques for critical ESD complications, such as perforation and postoperative stricture, have also been reported [1–7].

On the other hand, more basic problem is remained. After ESD, we decide whether curative resection or not based on the criteria for expanded indication which were defined from the retrospective study investigated 5265 patients who had undergone gastrectomy with lymph node dissection for early gastric cancer [8].

In the study, tumor size which was influenced by the force to extend a resected specimen was one of the most important factors of lymph nodes metastasis. Actually, in our long-term follow-up study of many ESD cases, we encountered several cases with regional lymph node recurrence despite endoscopic resection of early gastric cancer according to the expanded indications, and some other cases have been reported [9, 10].

To improve patient outcomes, we investigated whether the true tumor diameter of gastrointestinal cancer specimens measured by flexible endoscopy was subjective by testing if the specimen were correctly attached to a specimen board after ESD resection and whether the size differed depending on the endoscopist who attached the specimen.

## Materials and Methods

### Patients

Seventy-two patients diagnosed with early gastric cancer at Ehime Rosai Hospital and Kagawa University Hospital from January 31 to June 30, 2014 who satisfied the following expanded-indication inclusion criteria for ESD according to the guidelines of the Japan Gastric Cancer Association (JGCA) were included: In differentiated gastric cancers, intramucosal carcinoma has no size restriction, whereas intramucosal carcinoma accompanied by ulcer scar and SM1 carcinoma (500 μm from the muscularis mucosae) should be <30 mm. In undifferentiated carcinomas, intramucosal carcinoma should be <20 mm. Included patients consented to the study after receiving oral and written explanations.

The exclusion criterion was being diagnosed with beyond expanded indication for endoscopic resection before ESD according to the JGCA guidelines.

### Ethics statement

We confirmed that the clinical trial protocol we had included as S1 Protocol was the version that had been submitted to and approved by our ethics committee "The Ethics Committee of Ehime Rosai Hospital (No. 41) and Kagawa University" before the trial began.

Patients were provided informed consent by verbal and written forms and gave written consent for participation in this study.

### Trial registration

University hospital Medical Information Network (No. 000012915)

### Study design and protocol

Two endoscopists out of 10 engaged in the ESD were excluded. Three endoscopists were randomly extracted before every ESD by the envelope method among 8 endoscopists. Of these 3 endoscopists, each endoscopist extended the specimen evenly and stuck it to the specimen board with a setting pin, and measured the maximum resected diameter, and recorded it. Only Dr. H.K., who was informed of this study and never attached a resected specimen, removed the lesion from the board and handed the specimen to the next endoscopist in another room one by one.

Thus, the lesion was attached 3 times and recorded 3 times. All endoscopists attached a specimen and removed it as soon as possible to avoid drying effect affected to the specimen size. Moreover, during the attached and removal procedures, we immersed the specimen in the natural saline. The specimen attached by the third endoscopist was submitted for pathologic examination. The third endoscopist’s specimen became the pathology specimen.

**Fig 1:**
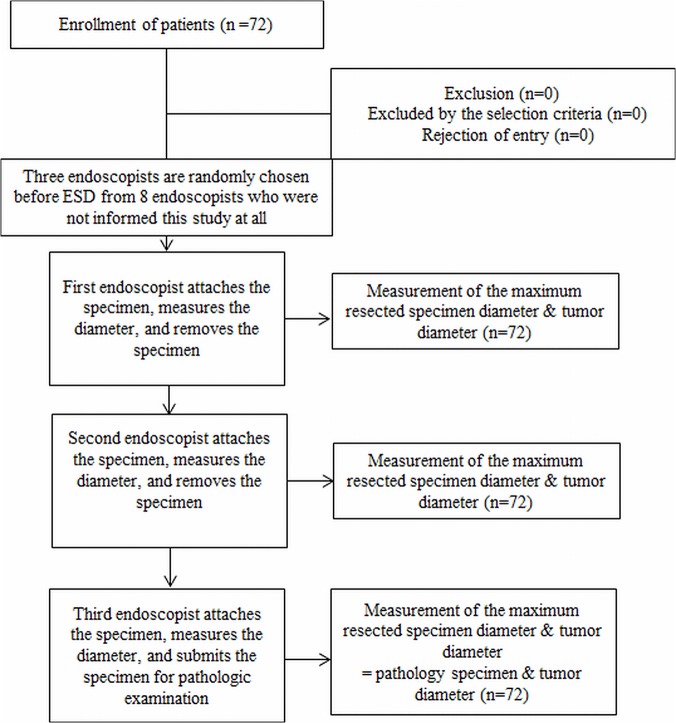
Flow chart of the prospective trial. Seventy-two patients diagnosed with early gastric cancer at Ehime Rosai Hospital and Kagawa University Hospital were included. From 10 endoscopists, the 2 endoscopists engaged in the ESD were excluded. Three endoscopists were randomly extracted before every ESD by the envelope method from among 8 endoscopists who were not informed of this study at all. These 3 endoscopists in turn attached the same resected lesion to a specimen board, measured the maximum resection diameter and tumor size, and recorded it.

We randomly selected only 3 endoscopists because more than 3 rounds of “attachment and removal” of 1 specimen led to damage its mucosa in 5 pilot studies **(Fig 1)**.

In our hospital, at first, as necessary pre-educations with regard to pathological techniques, all endoscopists were given instructions from their senior pathological instructors how to handle and attach the resected specimen naturally attached as it was. However, it were limitations for all senior pathological instructors to teach them more detailed instructions objectively such as degree of tension to the specimen **(S1 CONSORT Checklist) (S1 Protocol).**

### Sample size and final enrollment

We conducted 5 pilot studies, calculated the data (differences of resected specimen size and tumor size among 3 endoscopists) and statistically analyzed it is Graph Pad Prism 5 to obtain a sample size (n = 70) using G* power using the study power of 0.8, type I error level 0.05 (two tail t-test). http://biostat.mc.vanderbilt.edu/wiki/Main/PowerSampleSize

Finally, we enrolled 72 patients. The primary outcome was the difference in the maximum resection diameter of specimens and the recorded tumor size attached by the 3 randomly selected endoscopists.

### Statistical analysis

Data were analyzed using paired *t*-tests between 2 groups, and using Kruskal Wallis *H-* test among 3 groups. Multivariable analysis was conducted after adjustment for age, gender, tumor location (U/M/L) site, procedure time of ESD, resection diameter, lesion diameter respectively. The significance level was set at P<0.05. Statistical analyses were performed using Graph Pad Prism version 5 for Windows (Graph Pad Software, San Diego, CA, USA).

## Results

The 72 patients included 42 men and 30 women who were 47–88 years old (mean±standard deviation: 69.0±23.8). The lesion sites were in the upper region of the stomach (U), middle region (M), and lower region (L) regions (22, 24, and 26 patients). The ESD procedure time was 45–180 (105.6±23.6) min. In pathological results after ESD, numbers of histology (tub1/tub2/pap/por/sig/muc) were 49/18/3/1/1/0 respectively (Table 1). Although multivariable analysis was conducted after adjustment for age, gender, tumor location site (U/M/L), procedure time of ESD, resection diameter, lesion diameter respectively, there were no significant differences among these factors.

**Table 1:** Characteristics of patients.

No. patients	72
Gender (male/female)	42/30
Mean age±SD(range), years	69.0±23.8 (47–88)
Tumor location (U/M/L) (%)	22/ 24/26 (30.5%/33.3%/36.1%)
Procedure time of ESD (min)	105.6±23.6 (45–180)
Pathological findings (tub1/tub2/pap/por/sig/muc)	49/18/3/1/1/0

U; upper region, M; middle region, L; lower region of stomach

tub1; well differentiated tubular adenocarcinoma, tub2; moderately differentiated tubular adenocarcinoma, pap; papillary adenocarcinoma, por; poorly differentiated tubular adenocarcinoma, sig; signet-ring cell carcinoma, muc; mucinous adenocarcinoma

**Fig 2:**
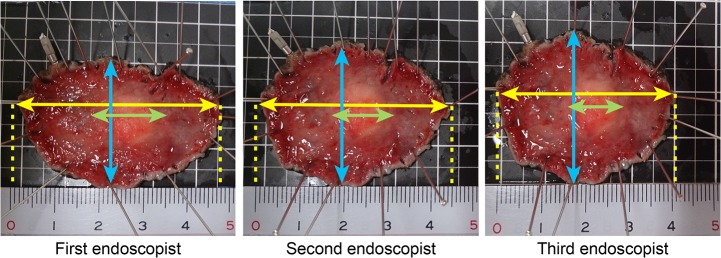
Photograph of the Case 27 lesion actually attached by 3 endoscopists. The maximum resection diameters shown by the yellow arrow are 48, 45, and 40 mm, showing variation. The orthogonal diameters shown by the blue arrows also vary. The tumor sizes (green arrows) were also different from each other.

A photograph of specimens attached by 3 endoscopists was shown for Case 27 **(Fig 2).** The maximum resection diameters were 48, 45, and 40 mm respectively, and the recorded tumor sizes were 17, 14, and 12 mm respectively, showing variation. The maximum resection diameter, the orthogonal diameter and the tumor size were varied among 3 endoscopists as those measurement results were depended on the difference in the extensive force and the direction of attachment.

**Fig 3:**
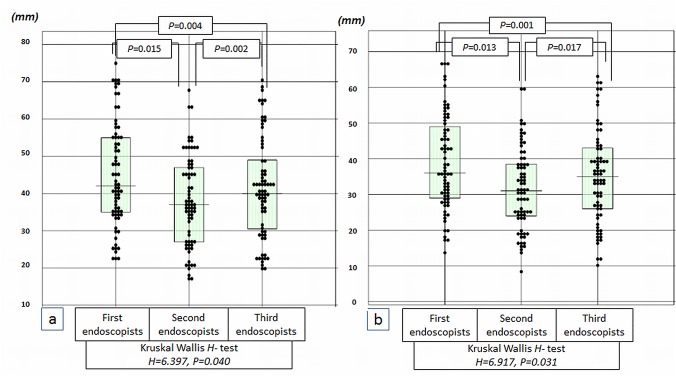
Comparison among 3 endoscopists. a: Comparisons of maximum resected specimen diameters among 3 groups resulted in significant differences (*Kruskal Wallis H- test*, *H = 6*.*397*, *P = 0*.*040*). b: Comparisons of tumor sizes among 3 groups resulted in significant differences (*H = 6*.*917*, *P = 0*.*031*).

The diameter of the third endoscopist was consistent with the diameter of the pathology specimen because infiltration of formalin caused no apparent reduction or other alteration of the specimen diameter. The resected specimen diameters of the 3 endoscopists were 44.5±13.9 mm (95% Confidence Intervals (CI): 23–67), 37.4±12.0 mm (95% CI: 18–60), and 41.1±13.3 mm (95% CI: 20–63) mm respectively. Comparison among 3 groups (Kruskal Wallis *H*- test), there were significant differences among 3 groups (*H* = 6.397, *P* = 0.040), and moreover, comparison between 2 groups (paired t-test) resulted in *P*-values of 0.015, 0.002, and 0.004 for the first-second, second-third, and first-third endoscopist pairs, showing significant differences (Fig 3A). The recorded tumor sizes in the first-second, second-third, and first-third endoscopist pairs were 38.3±13.1 mm (95% CI: 16–67), 31.1±11.2 mm (95% CI: 12.5–53.3), and 34.8±12.8 (95% CI: 11.5–62.3) mm. Comparison among 3 groups (Kruskal Wallis *H*- test), there were significant differences among 3 groups (*H* = 6.917, *P* = 0.031), and moreover, comparison between 2 groups resulted in *P*-values of 0.013, 0.017, and 0.001, respectively, showing significant differences (Fig 3B).

## Discussion

Accurate evaluation of the depth of early gastric cancer before treatment to apply the indication criteria of ESD remains difficult, even using endoscopic ultrasound and NBI magnified endoscopy. The criteria for expanded indication of intramucosal carcinoma appear to be adequate. These criteria are based on the study by Gotoda *et al* [8]. However, in our long-term follow-up study of many ESD cases, we encountered several cases with local lymph node recurrences despite endoscopic resection of early gastric cancer according to the expanded indications. Actually, some other cases have been reported [9, 10]. As long as metastasis-positive gastric mucosal cancers occurred more or less, this problem should be reconsidered. As there have been many evidence-based reports on histopathological and/or molecular biological studies [11–14], we needed to reconsider from another point of view.

As it might be impossible to preoperatively evaluate the invasion depth, lymph-vascular invasion, and vascular invasion of a lesion, we couldn’t help but evaluate the presence or absence of lymph node metastasis based on the lesion size [15]. Because pathologic examination including immunostaining is accurate, we endeavored to re-investigate the step in which human errors could occur. Because it is difficult to accurately measure the tumor diameter at the several-mm level with current endoscopic methods, the diameter is measured using an attached pathology specimen submitted as the post-resection specimen. We hypothesized that the step of preparing an attached specimen after endoscopic resection might result from human errors.

We couldn’t point out any significant difference between the pathological findings (tub1/tub2/pap/por/sig/muc 49/18/3/1/1/0 respectively) and these tumor sizes. Therefore, we presumed that the steps of preparing an attached specimen just after resection which individually depended on the extent force by each endoscopists might be caused the inaccuracy of the tumor size. Clinically, it’s very important that the objective process to attach the resected specimen before formalin fixation when the tumor size is change no longer in pathologically because the extended force used to extend a specimen naturally has individual differences.

In the present study, errors already occurred during the step in which the endoscopist attached a specimen of the same resected lesion. Therefore, with the current method of attaching a resected specimen, error due to the endoscopist who attaches the specimen is more or less unavoidable and should be treated very carefully. Particularly, in the case of undifferentiated gastric cancers <20 mm in diameter yet indicated for radical resection, evaluation of the size of the lesion is difficult because they progress discontinuously by invading beneath the mucosa [16].

The postoperative tumor diameter which caused no apparent reduction is a critical factor in pathological diagnosis [17, 18]. Over-evaluation leads to a recommendation for additional surgical resection and/or additional chemotherapy, whereas under-evaluation may lead to the local recurrence and/or lymph node metastasis.

**Fig 4:**
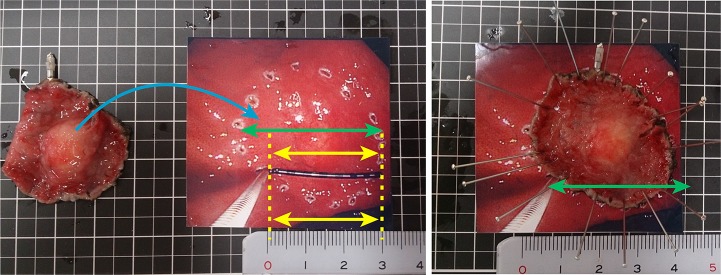
Equally magnified photograph attachment method. After the picture was taken in front of the lesion with measuring forceps to confirm that the photograph was equally magnified (yellow arrows). The lesion was photographed to record the size (green arrow indicates the tumor diameter) and shape of the lesion *in vivo* and printed. The resected specimen was attached as the photograph shows (blue curved arrow).

One of the more objective methods was shown (Fig 4). After the picture was taken in front of the lesion just after marking for ESD with measuring forceps to record the size of the lesion *in vivo*, the picture was printed at equal magnification by checking the size of the measuring forceps in the picture. If the picture was not the same size, a copy was enlarged or reduced to the same size. The outside of the marking of the lesion was resected for attachment as the photograph shows. If the lesion cannot be viewed from the front, it lacks objectivity. A more objective evaluation method is urgently required. Nonetheless, this method is still more objective than attachment of the resected lesion by only 1 endoscopist. This study has several limitations such as inter-observer variability and any effect of drying on the specimen size pre-fixation. There was significant difference between inter- endoscopists with regard to tumor size. As we conducted multivariable analysis after adjustment for several factors, there were no significant differences among these factors.

In conclusion, human errors regarding the size of attached resected specimens that affect the patient’s additional treatment or even surgical intervention are unavoidable and cannot be ignored because it depend on more individual factors. We must develop more precise methodology to obtain accurate tumor size.

## Supporting Information

S1 CONSORT Checklist This CONSORT checklist include each section of this clinical prospective trial. (PDF)

S1 Protocol This application form is Original Protocol and Ethical Review of the Ethics Committee of Ehime Rosai Hospital and Kagawa University Hospital. (PDF)
