# Supplementary material for: Unavoidable Human Errors of Tumor Size Measurement during Specimen Attachment after Endoscopic Resection: A Clinical Prospective Study
Source: PLoS One. 2015 Apr 9;10(4):e0121798. doi: 10.1371/journal.pone.0121798 (PMC4391867; doi:10.1371/journal.pone.0121798)
Supplement: S1 Protocol — (PDF) [file pone.0121798.s002.pdf]

Reception number: 41

Protocol and Ethical Review Application Form

Submission Date: January 10, 2014

To:
The Chairperson of the Clinical Ethics Committee of Labor Health and Welfare Organization, Ehime Rosai Hospital

Applicant, Affiliation:
Hirohito Mori
Department of Gastroenterology and Neurology, Kagawa University, 1750-1 Ikenobe, Miki, Kita, Kagawa 761-0793, Japan
Department of Gastroenterological Surgery, Ehime Rosai Hospital, 13-27, Minamikomatsubara, Niihama, Ehime 792-8550, Japan

1. The name of the clinical study

Unavoidable human error in tumor sizing during attachment of resected specimens after endoscopic resection: a randomized prospective study

2. The manager of the study, Affiliation

Hirohito Mori
Department of Gastroenterology and Neurology, Kagawa University, 1750-1 Ikenobe, Miki, Kita, Kagawa 761-0793, Japan
Department of Gastroenterological Surgery, Ehime Rosai Hospital, 13-27, Minamikomatsubara, Niihama, Ehime 792-8550, Japan

3. The collaborator and contributor of the study, Affiliation

Takaaki Tsushimi
Department of Gastroenterological Surgery, Ehime Rosai Hospital, 13-27, Minamikomatsubara, Niihama, Ehime 792-8550, Japan
Hideki Kobara, Noriko Nishiyama, Shintaro Fujihara, Tsutomu Masaki
Department of Gastroenterology and Neurology, Kagawa University, 1750-1 Ikenobe, Miki, Kita, Kagawa 761-0793, Japan

4. Aim

Objective evaluation of resected specimens and tumor size is critical because the tumor diameter after endoscopic submucosal dissection (ESD) affects therapeutic strategies. In this study, we investigated whether the true tumor diameter of gastrointestinal cancer specimens measured by flexible endoscopy is subjective by testing if the specimen is correctly attached to a specimen board after ESD resection and whether the size differs depending on the endoscopist who attached the specimen.

5. Subjects of this research and study

Patients who are diagnosed with early gastric cancer satisfied expanded-indication guideline for ESD of the Japan Gastric Cancer Association (JGCA). JGCA defined the expanded-indication inclusion criteria of ESD as follows: In differentiated gastric cancers, intramucosal carcinoma has no size restriction, whereas intramucosal carcinomas accompanied by ulcer scarring and SM1 carcinomas (500μm from the muscularis mucosae) should be <30 mm. In undifferentiated carcinomas, intramucosal carcinomas should be <20 mm. Included patients provided consent after receiving oral and written explanations of the study. The exclusion criterion was being diagnosed with a lesion with off-label indications for endoscopic resection before ESD according to the JGCA guidelines.

In this study, to improve patient outcomes, we investigated whether the true tumor diameters of gastrointestinal cancer specimens measured by flexible endoscopy are subjective by testing if the specimen is correctly attached to a specimen board after ESD resection and whether the size differs depending on the endoscopist who attached the specimen.

6. Implementation location

Kagawa University Hospital, 1750-1 Ikenobe, Miki, Kita, Kagawa 761-0793, Japan

Ehime Rosai Hospital, 13-27, Minamikomatsubara, Niihama, Ehime 792-8550, Japan

7. Implementation plan

From among ten endoscopists, the two endoscopists engaged in the ESD were excluded. Three endoscopists were randomly selected before every ESD by the envelope method from eight endoscopists who were not informed of this study at all. These three endoscopists in turn attached the same resected lesion to a specimen board, measured the maximum resection diameter and tumor size, and recorded it. Therefore, the lesion was attached three times and recorded three times. The specimen attached by the third endoscopist was submitted for pathological examination. The third endoscopist’s specimen became the pathology specimen.

The primary outcomes were the difference in the maximum resection diameter of the specimens and the recorded tumor size attached by the three randomly selected endoscopists.

8. Possibilities of disadvantage and risk of individual health due to this research and study

After conventional ESD by an ESD expert, each specimen is attached and removed from the board and handed to the next endoscopist in another room three times. Therefore, there is

no direct influence on the patient.
The specimen attached by the third endoscopist will be submitted for pathologic examination. Third endoscopist's specimen becomes the pathology specimen. Therefore, there is no possibility of disadvantage or risk to individual health.

9. Medical contribution of this research and study

It is impossible to preoperatively evaluate the invasion depth, lymphovascular invasion, and vascular invasion of a lesion, but we cannot help but evaluate the presence or absence of lymph node metastasis based on lesion size. Because pathological examination including immunostaining is accurate, we endeavored to re-investigate the step in which error could occur. Because it is difficult to accurately measure the tumor diameter at the several-mm level with current endoscopic methods, the diameter is measured using an attached pathology specimen submitted as the post-resection specimen. More accurate pathological specimen sizing will be obtained.

10. Protection of individual human right and informed consent during this research and study

Patients will be provided informed consent by verbal and written forms.
The ethical protection of individual human rights are conducted according to the Helsinki Declaration.
① Protection of privacy and individual information.
All researchers in this study have to handle patient privacy and individual information sufficiently and protect it adequately.
Moreover, no researcher will be allowed to publish any data or information that might identify the patients.
② Method of handling patient information and identification of researchers.
All patient names are encoded, and the correspondence table between the codes and patients is provided a safe management system in Ehime Rosai Hospital and Kagawa University Hospital.
All correspondence tables between codes and patients will have to be deleted and permanently disposed of.

11. Other

Trial registration

University hospital Medical Information Network (No. 000012915)
